# De Voce, Lingua, Respiratione, Deglutitione Observationes Quædam

**Published:** 1844-05

**Authors:** 


ART. VII.—*De Voce, Lingua, Respiratione, Deglutitione Observationes quædam.* Dissertatio Inauguralis Physiologica, quam ex auctoritate et consensu gratiosi Medicorum Ordinis in alma litterarum Universitate Fredericia Gulielmia Rhenana, ad Doctoris Medicinæ et Chirurgiæ gradum rite assequendum, scripsit et die v. Aprilis anni MDCCCXXXXI publice defendit CAROLUS ERNESTUS NOEGGERATH, Rhenano-Borussus. Cum tabula lithographica. Bonnæ, typis Caroli Georgii, MDCCCXLI.

*Observations on Voice, Speech, Respiration, and Deglutition:* being an Inaugural Dissertation presented to the Medical Faculty of the Frederic-William University at Bonn, on the 5th of April, 1841, for the Degree of Doctor of Medicine and Surgery. By CHARLES ERNEST NOEGGERATH. With a lithograph. Bonn: pp. 14, 4to. Charles George: 1841.

The observations and experiments detailed in this paper, were made upon an individual with a fistulous opening in the anterior part of the neck. The author remarks that although wounds in this region are not uncommon (as in attempts at suicide) yet very little of benefit to physiology has resulted from them, owing either to the sudden death of the person, or to the immediate closure of the wound by the surgeon, and its subsequent complete cicatrization. It is true, some examples of fistula are on record: thus Mayo relates two, Dupuytren a third, and our author quotes one from Cornelius Trioen and gives another in the course of his observations from Froirep's *Notizen* for 1831. But in all these cases, except the last, the fistula was below the epiglottis.

In our notice of Dr. Noeggerath's Thesis, we shall follow the order he has laid down. As the case of injury is altogether a singular one we shall present his account of it with but little alteration.

The individual, Christian Mueller, is a native of Heimbach, in Prussia, and was born in 1799. At the age of 16 he turned soldier. He seems to have been a pretty quarrelsome fellow, and as brave as quarrelsome, for he was engaged in several duels, which were generally decided by the small sword. He was frequently wounded in these engagements, and in one of them received a thrust in the chest which injured the lung, and confined him 66 days. At length, after various fortunes, he found himself in his 30th year, at the head of a troop of horse in Africa. In the expedition against Constantine, his horse was struck by a bullet and fell with him. Whilst lying on the ground, Mueller received a cut from the yatagan of an Arab, which pierced his silk cravat, and the collar of his shirt, and divided the pharynx above the epiglottis and thyroid cartilage, leaving, however, the posterior wall untouched. Blood flowed profusely, which he checked in some measure by filling the wound with the divided shirt; his strength failed rapidly and he had vomitings of blood, &c. At the moment he was wounded he felt no pain, nor until some minutes had elapsed, when he experienced severe burning pain and intolerable thirst. After the wound was dressed, he was carried to Algiers in a state of insensibility; traumatic delirium then came on, and continued for seventeen days, during which time he took immense quantities of opium, enough to kill a horse, as the surgeons remarked, all without effect. The greater part, it was supposed, passed through the wound. After this he was near starving to death, as food of all kinds passed through the fistulous opening of the throat. At length, however, after suffering all the tortures of Tantalus, and after the surgeons had tried in vain to close the fistula by plates of silver and lead, the patient himself adapted to the opening a piece of leather covered with linen, which by its elasticity supplied, in some measure, the divided parts. He left the hospital on the 27th day, at first quite weak, but was by and by able to resume his military duties. He remained in Africa two years longer, and subsequently went to Paris where Jobert cured him of hydrocele.

Mueller is now (at the time of the experiments) 41 years old, five feet seven inches high, and well formed. He stoops some in walking, and shows some lassitude, but can make a long journey without much fatigue. His chest is broad, and sounds well on ausculting and percussing it. Comparing his present state with that previous to the wound, he says he does not feel so vigorous, that his voice is not so strong, and that he does not, as formerly, require occasional bleeding on account of constriction and oppression of the chest.

The aperture presents the following appearances: When he breaths quietly, with the neck extended naturally, the aperture is one inch ten lines and a half long, and one inch and one line broad. In the superior part of the opening and anteriorly, the root of the tongue is seen, covered apparently with granulations; between this and the posterior part of the fauces, a little above, are placed the posterior half-arches of the palate, the uvula depending from their apex. Internally is seen the epiglottis nine lines long, erect, but turned slightly to the left; the vessels of the surface are injected, so that it is a little redder than natural, but less so than the posterior wall of the pharynx. It is generally contracted, owing to the flow of mucus over the surface; the perichondrium and mucous coat a little thicker than natural. In the lower margin of the opening a hard tubercle can be felt, formed by the base of the os hyoides, which is closely united to the thyroid cartilage. The greater cornua of the os hyoides are seen at the sides. The space between the thyroid and cricoid is natural, but the whole larynx is so much depressed that the superior part of the cricoid is only seven lines above the sternum. The parts injured, besides the common integuments, are the platysma myoid muscle and the subcutaneous nerves, the mylo-hyoid, genio-hyoid, and part of the hyoglossus. The ranine artery was doubtless divided, from the profuse hemorrhage at the time of the injury. The sensibility of the injured parts appears very much diminished, as they can be touched with the finger, and the individual himself cleanses them with lint, without the production of pain. Strong pressure finally excites vomiting. The difference, however slight, in the temperature of ingested liquids, is perceived. The skin of the infra-mental region exhibited so little sensibility that Prof. Muller thought a portion of it might be removed without the patient's feeling it.

We do not know that the experiments instituted by Dr. Noeggerath present any new views on the subjects to which they refer; but they confirm certain theories and notions, and effectually subvert others that are strenuously upheld by certain physiologists. We shall present a pretty full account of them.

I. *Observations and Experiments on the Voice.* When the fistula was open Mueller's voice, subraucous, could be distinctly heard, showing that the voice exists deep in the pharynx and in the rima glottidis. The glottis itself, and the ligaments, owing to their anatomical seat, could scarcely be seen. Nevertheless professor Mayer declared positively that he looked into the glottis itself, during the production of voice, and observed the erection of the arytenoid cartilages, and the tension and tremulation of the vocal ligaments, though the tension could not be distinguished clearly. This accords with the experiments of sundry physiologists on artificial larynges.

When the fistula was closed, the voice was stronger; showing that the cavity of the pharynx, mouth, nose and the adjacent cavities increase the voice not a little. This is easily explained upon physical laws, supposing the effect of the superior parts to be similar to that of a tube; for Biot has shown that hollow but dense conductors increase the force of sounds by hindering the dissipation of the rays. The action of the stethscope illustrates the same principle. This was well understood by Haller and Santorinus.

When the fistula was open the voice was less sonorous. This agrees with Prof. Müller's conjecture, that the circumstance of the vocal tube's having a double orifice, the oral and nasal, does not affect the height of the notes any more than a simple tube; it changes their character by resonance, the voice being of the same altitude, but rendered more sonorous.

These last observations are confirmed by the experiments of Bennati, Savart, and Cagniard la Tour, on one Philibert Hercolot, who wounded himself in the neck with a knife; the incision, at first four inches in extent, was reduced in three months to a fistula of two or three lines between the thyroid cartilage and the hyoid bone.

When very loud sounds were uttered, the epiglottis was inclined forwards, turning a little from the lateral into a more horizontal position; at the same time it seemed more tense, though a tremor was perceptible by the finger. This confirms an observation of Mayer. "The ancients," he says, "aptly compared the epiglottis to an ivy leaf. By rolling back on itself, it receives the voice coming from the rima glottidis into a very narrow channel, collects the various rays of sound, dispersing them toward the middle of the canal. This infraction is the greater as the epiglottis is more extended. On the other hand, it continues erect in the hoarser sounds, and does not prevent their dissipation."

Touching the epiglottis while the man was speaking, rendered the voice weaker, deeper and hoarser, owing doubtless to the abnormal plane and impeded vibrations of the epiglottis. This seems to confirm Müller's views of the uses of this structure, and those of Magendie drawn from Grenié's experiments.

When he uttered very loud sounds, and at the same time raised the voice, a vehement commotion of the air was distinctly perceived by the finger inserted in the fistula. This again accords with the observations of Liscovius, Lehfeldt, and Müller, from which it appears that an increase of the force of the current of air, other things being equal, raises the pitch both of the natural and falsetto notes.

He sang well the whole scale of sounds whether the fistula was open or closed; from which it follows that they are formed above the pharnyx. According to Rudolphi singing is performed by the larynx alone, as seen in birds, and hence such as use the lips and mouth make hissing sounds. In singing the notes were not pleasant and agreeable, and the man was easily fatigued. He says that before being wounded, he could not utter higher notes than he now does. In the case of Hercolot, previously mentioned, it was otherwise.

II. *Observations and Experiments on Speech.* When the fistula was covered. Mueller could utter very distinctly all the letters and any name whatever, in a clear voice. Nor was there much alteration of voice, whether the aperture was closed by wood, glass, leather, or a metallic substance. When the fistula was open he could utter distinctly but one vowel letter, *a* with a slight admixture of *e*. He sounded *i* least of all. Hence it follows that in forming the vowels, without some greater difference of sound in the larynx, the greatest and most important differences are produced in the pharynx and cavity of the mouth. Müller, indeed, asserts that in pronouncing the vowels either aloud or in a whisper, the sound is formed by the larynx but is changed in the mouth to *a*, *e*, *i*, *o*, *u*. Is not this sound, though, called in question by Müller, from its quality, that of *a* or its analogue? and does it not seem that the infantile names of parents—mama, papa, *vater*, *pater*, father—in which the *a* constantly prevails in all nations, are derived from the period of life, in which the action of the mouth and palate is imperfect?

When the patient tried to pronounce *a*, *o* and *u*, the epiglottis remained plane, but in pronouncing *e* and *i* it was turned a little on itself.

Of the consonants, he could pronounce very well *h*, and when he laughed, *r*. This proves that *r* belongs to the glottis. Most physiologists are silent concerning this letter. According to Haller it is produced mainly by the tongue; Lenhossek expresses the same opinion. Müller speaks of two r's, pure or lingual, and palatal. Mayer, however, correctly says: "R belongs to the glottis, and is produced partly by the vibration of the arytenoid cartilages and partly by that of the glottis: it is the sound singers make in producing the shake (*tremulatio*)."

III. *Observations and Experiments on respiration.* In respiring with the fistula open, fatigue was soon induced; and respiration was three times more frequent than when the opening was closed, it being then twenty to the minute. This fatigue and increased frequency of respiration, arose, Noeggerath thinks, from two causes: 1. That the air thus introduced was colder than if it had passed through the buccal and nasal cavities, and hence acted as an irritant: 2. That by its quantity, and perhaps also by its quality, it was little suited to the purposes of respiration. As it respects quantity, it is observed that if one breathes through the mouth alone, the nose being closed, he soon becomes fatigued, and either dilates the mouth still more, or breathes more frequently in the same space of time. The air, moreover, before entering the larynx, is changed in other respects, besides that of heat, by contact with the mucous membranes. At all events it is evident that the watery particles are increased by its passing through the cavities of the nose and mouth, and that small particles of dust mechanically admixed are thus removed. In inspiration the larynx ascended, in expiration it descended. When the breath was held a long time, the epiglottis fell down upon the larynx, as if to close it mechanically.

Liscovius contends that the epiglottis, when at rest, holds the mean between the highest and lowest position. This is not confirmed by Mueller's case; in him, when the epiglottis was quiescent it was much more raised; nevertheless, the position of the hyoid bone should be considered, as upon it depends that of the epiglottis.

According to Mayo, the larynx has nothing to do with *snoring*, and most physiologists, like Lenhossek, attribute it to the vibration of the velum. Yet in the wounded man, when the fistula was open, an obscure stertorous noise was produced, showing that the larynx was engaged in it.

IV. The last experiments and observations refer to *deglutition*

The wound being covered with a bandage, liquids could be swallowed easily, solid food a little less so, and dry substances, as bread, better than those saturated with liquids, *e. g.* bread steeped in coffee. This is what occurs in all persons, except where there is idiosyncrasy or disease.

Frequently in swallowing liquids, especially a large quantity at once, the fistula being open, cough was produced by a portion falling into the glottis. This seemed to be owing to the fact that the glottis, on account of the muscles being cut, was not sufficiently raised, and was obliquely covered by the epiglottis.

In the effort to swallow, the larynx was drawn up, and the pharynx contracted by the palato-pharyngeus, so that the epiglottis was depressed upon the larynx. This happened before the descent of the bolus, which, therefore, does not mechanically depress the epiglottis.

When the fistula was uncovered, the saliva constantly flowed over the posterior parietes of the pharynx; whether more than when closed, it is impossible to say, although such seemed to be the case. When the covering was removed, the flow of saliva increased, the colder air in the larynx irritating the organs by contact or by sympathy. Rudolphie holds the opinion that the saliva is constantly secreted, but Muller says that when the muscles concerned in mastication, and the tongue, are completely at rest, and there is no unusual stimulus acting upon the nerves, the secretion ceases, whilst it is excited under the opposite circumstances. In the present case, a fistula of four years' standing could hardly be regarded as an unwonted nervous irritant, nor could the salivary flux depend upon injury of the salivary organs.

It was only occasionally that the patient could swallow a bolus when the fistula was open. Dr. N. attempted to close it by plates of glass, varying in size and form; but without success. An elastic body was requisite, which, insinuating itself into the wound, and imitating the motions of the divided parts, impeded neither the movements of the fauces nor larynx.

Lastly, some experiments on *taste* were instituted by Dr. Kobelt. When tincture of colocynth was applied to the epiglottis and pharynx, no sensation followed; when it was applied to the surface of the velum and uvula, a bitter taste was by and by perceived. But as the velum is touched by the root of the tongue every moment, the experimenter did not feel warranted in making a conjecture as to the patient's sense of taste.

We have thus given a rapid and free translation of the most important facts in Dr. Noeggerath's thesis; we leave the reader to examine, where the author has not done it, how far they agree or disagree with received physiological notions. When we say *rapid* translation, we do not mean to give the impression that it was quite as easy as writing one's vernacular. In fact, it is in some places a decidedly crabbed specimen of latinity, notwithstanding its production at one of the most celebrated Prussian and European Universities. It contains numerous errors of the press, and some egregious ones, besides, of a kind that cannot be attributed to printers and careless proof-readers. For example, we mistake very much if *vulnus* forms a genitive plural, *vulnerorum;* yet this is the fourth word in the thesis. Again, the author construes *sonus* both in the second and fourth declensions, more frequently, perhaps, in the latter, yet it rightfully belongs to the second, and to the second only. This mistake we think the less pardonable, as it was at first the source of some perplexity; and either of them is richly deserving of the birch. We shall not mention any others, and we speak of these with the modesty that should characterize one who acquired his smattering of Latin, not in college halls and bowers, but in the silence and solitude of his own chamber—one not too luxuriously furnished, but which looked forth on as fair a prospect as ever met the gaze of mortal man.

Dr. Noeggerath has, we believe, taken up his residence at Mills' Point, in this State. We trust that his new home will prove a pleasant one, that his success will be commensurate with his undoubted learning and skill, and that his most glowing dreams, such as he may have cherished on the banks of the "wide and winding Rhine," will be more than realized on the shores of the Mississippi.

